# Parametric and Nonparametric Population Pharmacokinetic Models to Assess Probability of Target Attainment of Imipenem Concentrations in Critically Ill Patients

**DOI:** 10.3390/pharmaceutics13122170

**Published:** 2021-12-16

**Authors:** Femke de Velde, Brenda C. M. de Winter, Michael N. Neely, Jan Strojil, Walter M. Yamada, Stephan Harbarth, Angela Huttner, Teun van Gelder, Birgit C. P. Koch, Anouk E. Muller, on behalf of the COMBACTE-NET Consortium

**Affiliations:** 1 Department of Medical Microbiology and Infectious Diseases, Erasmus University Medical Center, 3000 CA Rotterdam, The Netherlands; anoukemuller@gmail.com; 2 Department of Hospital Pharmacy, Erasmus University Medical Center, 3000 CA Rotterdam, The Netherlands; b.dewinter@erasmusmc.nl (B.C.M.d.W.); t.vangelder@erasmusmc.nl (T.v.G.); B.koch@erasmusmc.nl (B.C.P.K.); 3 Laboratory of Applied Pharmacokinetics, Keck School of Medicine, University of Southern California, Los Angeles, CA 90027, USA; mneely@chla.usc.edu (M.N.N.); wyamada@chla.usc.edu (W.M.Y.); 4 Department of Pharmacology, Palacky University, CZ-779 00 Olomouc, Czech Republic; jan.strojil@upol.cz; 5 Division of Infectious Diseases, Faculty of Medicine, Geneva University Hospitals, 1205 Geneva, Switzerland; stephan.harbarth@hcuge.ch (S.H.); Angela.Huttner@hcuge.ch (A.H.); 6 Infection Control Program, Faculty of Medicine, Geneva University Hospitals, 1205 Geneva, Switzerland; 7 Department of Medical Microbiology, Haaglanden Medical Centre, 2501 CK The Hague, The Netherlands

**Keywords:** imipenem, population pharmacokinetic modeling, parametric, nonparametric, simulations

## Abstract

Population pharmacokinetic modeling and simulation (M&S) are used to improve antibiotic dosing. Little is known about the differences in parametric and nonparametric M&S. Our objectives were to compare (1) the external validation of parametric and nonparametric models of imipenem in critically ill patients and (2) the probability of target attainment (PTA) calculations using simulations of both models. The M&S software used was NONMEM 7.2 (parametric) and Pmetrics 1.5.2 (nonparametric). The external predictive performance of both models was adequate for eGFRs ≥ 78 mL/min but insufficient for lower eGFRs, indicating that the models (developed using a population with eGFR ≥ 60 mL/min) could not be extrapolated to lower eGFRs. Simulations were performed for three dosing regimens and three eGFRs (90, 120, 150 mL/min). Fifty percent of the PTA results were similar for both models, while for the other 50% the nonparametric model resulted in lower MICs. This was explained by a higher estimated between-subject variability of the nonparametric model. Simulations indicated that 1000 mg q6h is suitable to reach MICs of 2 mg/L for eGFRs of 90–120 mL/min. For MICs of 4 mg/L and for higher eGFRs, dosing recommendations are missing due to largely different PTA values per model. The consequences of the different modeling approaches in clinical practice should be further investigated.

## 1. Introduction

Population pharmacokinetic (popPK) modeling and simulation is used to improve antibiotic dosing and clinical outcomes of infections. Antimicrobial efficacy is determined by the susceptibility of the drug in vitro (usually expressed as the minimal inhibitory concentration, MIC) and the exposure to the drug in vivo, which relies on the pharmacokinetics and the dose [1]. PopPK models describe the variability of exposure to a drug, and are therefore used to support dosing optimization. This optimization can take place in different ways: individualization of dosing via therapeutic drug monitoring (TDM) software, improving dosing regimens from the package insert (especially for specific subpopulations), and setting clinical breakpoints on a population level [2]. Clinical breakpoints are MICs that categorize microorganisms as susceptible or resistant to specific antibiotics [3]. 

Several popPK modeling methods are available. Statistically, they are classified as either parametric or nonparametric methods [2]. Parametric methods assume that the population parameter distribution is known, with unknown population parameter estimates [4]. Nonparametric methods make no assumption about the shapes of the underlying parameter distributions, by which, theoretically, subpopulations are more easily detected [5]. Many parametric and nonparametric popPK models are published in the literature, often accompanied by simulations of the model which lead to dosing recommendations [2]. Little is known about the differences in modeling and simulation results between parametric and nonparametric methods, which may influence dosing recommendations. 

Previously, we described the development and results of parametric and nonparametric popPK models of imipenem in critically ill patients [6]. Both models described imipenem popPK well, and the population parameter estimates were similar. The same covariate was included: the CKD-EPI (Chronic Kidney Disease Epidemiology Collaboration) eGFR (estimated Glomerular Filtration Range) equation [7], which was unadjusted for body surface area, on elimination rate K_e_. The estimated between-subject variability (BSV) was higher in the nonparametric model. External validation and simulations of both models were not yet performed.

Like other beta-lactams, the antibacterial effect of imipenem is determined by the percent of time of the dosing interval during which the free concentration remains above the MIC (*f*T _> MIC_) [8]. Reported targets for beta-lactam antibiotics range from 20 to 100% *f*T _> MIC_ to 100% *f*T _> 5xMIC_ [9,10,11,12,13]. Smaller preclinical and clinical studies suggest that the required targets seem to be the highest in cephalosporines, followed by penicillins, and then carbapenems [11,14]. Other M&S studies of imipenem in critically ill patients used targets of 20–100% *f*T _> MIC_ [15,16,17]. The DALI study showed a significant association of positive clinical outcome (defined as no switch of addition of antibiotics needed) with 50% *f*T _> MIC_ (OR 1.02) and 100% *f*T _> MIC_ (OR 1.56) for eight beta-lactams in 361 critically ill patients [18]. However, this study did not distinguish between the three classes of beta-lactams. Due to the lack of consensus about the target, we chose to use two targets in this paper (50% *f*T _> MIC_ and 100% *f*T _> MIC_). A Swiss study in hospitalized patients treated with standard imipenem dosing regimens from the package insert found a trend towards increased clinical failure in case of trough levels < 2 mg/L (11% vs. 19%), indicating that the dosing could be optimized. Unfortunately, this study was underpowered to detect a significant difference [19].

The first objective of the current study was to determine which of the two previously described imipenem models [6] delivers the best Bayesian posterior estimates to predict the imipenem concentrations in an external independent database. The second objective was to determine the probability of target attainment (PTA) for several doses and estimated glomerular filtration rate (eGFR) values using simulations of both models.

## 2. Materials and Methods

### 2.1. Population PK Models

Two previously published parametric (using NONMEM 7.2) and nonparametric (using Pmetrics 1.5.2) population PK models of imipenem in critically ill patients were used for the analyses in this paper. The development and results of both models are described in detail elsewhere [6]. Both models included two distribution compartments and the absolute (unadjusted for body surface area) CKD-EPI eGFR [7] as a covariate on the elimination rate constant (K_e_). The parameter estimates in both models were comparable, except from the estimated BSV, which was higher in the nonparametric model. The parameter estimates are displayed in Supplementary Table S1.

### 2.2. Population Used for Modeling

The models were built using imipenem PK data of 26 critically ill patients from a previously published prospective cohort study [20] in the intensive care unit (ICU) of the Geneva University Hospitals (Geneva, Switzerland). Inclusion criteria were suspected or documented severe bacterial infection and age between 18 and 60 years. Exclusion criteria were estimated glomerular filtration rate (eGFR) < 60 mL/min (measured by the Cockcroft–Gault equation [21]), Body Mass Index (BMI) < 18 or >30 kg/m^2^, and pregnancy. None of the patients received continuous renal replacement therapy (CRRT). None of the patients used probenecid, which is the only drug that is known to influence imipenem concentrations [22]. The usual dosing regimen for imipenem/cilastatin was 500 mg/500 mg every 6 h, administered by intermittent intravenous infusion for 30 min. 

Peak (approximately 15–30 min after end of infusion), intermediate (midway between two sequential administrations), and trough (approximately 15 min before the next dose) blood samples (*n* = 138) were collected on days 1, 2, 3, 4, and/or 6 of therapy; 47% was drawn on the second day. After centrifugation of the blood, MOPS [3-(*N*-morpholino)propanesulfonic acid], a stabilizing buffer that protects imipenem from degradation [23], was added to an equivalent volume of plasma. Imipenem plasma concentrations were analysed by high-performance liquid chromatography (HPLC), with ultraviolet (UV) detection at 298 nm. A median of three creatinine measures per patient were available.

Fewer than 10% [24] of all concentrations (13/138 = 9.4%) were below the limit of quantification (0.5 mg/L) and were excluded from the popPK analysis. All concentrations above the LOQ (*n* = 125) were included for popPK analysis.

### 2.3. Population Used for Validation

The external dataset consisted of imipenem PK data of 19 critically ill patients from a previously published prospective randomized study [25] in the ICU of the General University Hospital (Prague, Czech Republic). Inclusion criteria were hospital acquired pneumoniae (HAP) and age above 18 years. Exclusion criteria were carbapenem allergy, hepatic dysfunction (total serum bilirubin > 27 μmol/L), neutropenia (granulocytes < 500/mm^3^), acute or chronic renal failure (serum creatinine > 280 μmol/L or CRRT), obesity (BMI > 35 kg/m^2^ or weight > 110 kg), and pregnancy. None of the patients used probenecid.

Patients were randomized to receive either short infusion (bolus group) or extended infusion (extended group) of imipenem/cilastatin. Patients in the bolus group received 1 g/1 g imipenem/cilastatin every 8 h, administered by intermittent intravenous infusion for 30 min. Patients in the extended group received an initial loading dose of 1 g/1 g imipenem/cilastatin over 30 min, followed by an infusion of 500 mg/500 mg imipenem/cilastatin administered over 3 h every 6 h.

Blood samples (*n* = 114) were drawn on the second day of therapy: one sample prior to infusion and then at 0.33, 0.67, 4, 6, and 8 h (bolus group) or 2, 3.17, 4, 5, and 6 h (extended group). After centrifugation, MOPS buffer was added to an equivalent volume of plasma. Imipenem plasma concentrations were analysed by HPLC-UV at 313 nm. One creatinine measure per patient was available.

Fewer than 10% [24] of all concentrations (3/114 = 2.6%) were below the limit of quantification (0.26 mg/L) and were excluded from analysis. All concentrations above the LOQ (*n* = 111) were included for analysis.

### 2.4. External Validation

Imipenem concentrations of the external validation database were predicted using the parametric and nonparametric models. Subsequently, the prediction errors (individual predicted concentration minus observed concentration) and relative prediction errors (prediction error/observed concentration) were calculated. The prediction errors were also calculated using Monte Carlo simulations (*n* = 1000) of both models.

To visualize the external validation, plots with predicted versus observed concentrations and visual predictive checks (VPCs) were generated. For each VPC, a set of 1000 simulated datasets (using one of the popPK models developed with the modeling population) was created to compare the observed concentrations of the external validation database with the distribution of the simulated concentrations. Stratification on dose (500 mg and 1000 mg) and eGFR (measured by the CKD-EPI equation unadjusted for BSA) was applied. For eGFR, stratification in three groups (19–46, 50–89, and 90–178 mL/min) and two groups (19–59 and 79–178 mL/min) was performed. These ranges were chosen to create equal groups.

### 2.5. Simulations

Monte Carlo simulations were performed using the final models. The three imipenem dosing regimens from the package insert [22,26] were evaluated: 500 mg every 6 h (q6h), 1000 mg every 8 h (q8h), and 1000 mg q6h, each for a predefined eGFR (measured by the CKD-EPI equation unadjusted for BSA) of 150, 120, and 90 mL/min. The infusion rate was 1000 mg/h for each dosing regimen. Five thousand subjects were simulated for each combination of dosing regimen and eGFR. For each simulated concentration–time profile, the *f*T _> MIC_ was calculated for MICs of 0.015–64 mg/L. The unbound imipenem concentrations were calculated from the total concentration using a fixed value for protein binding of 20% [22]. Subsequently, the probability of target attainment (PTA) for 50% and 100% *f*T _> MIC_ was calculated. A PTA threshold of 97.5% [3] was chosen.

### 2.6. Software

Parametric population PK modeling and simulation was performed using NONMEM (version 7.2, ICON Development Solutions, Ellicott City, MD, USA), Intel Visual Fortran Compiler XE 14.0 (Santa Clara, CA, USA), RStudio (version 1.1.456; RStudio, Boston, MA, USA, 2018), R (version 3.5.1; R foundation, Vienna, Austria, 2018), XPose (version 4.6.1; Uppsala University, Department of Pharmaceutical Biosciences, Uppsala, Sweden, 2018), PsN (version 4.6.0; Uppsala University, Department of Pharmaceutical Biosciences, Uppsala, Sweden, 2016), and Pirana [27] (version 2.9.4; Certara, Princeton, NJ, USA, 2018). The *f*T _> MIC_ and PTA were calculated using Excel 2013.

Nonparametric population PK modeling, simulation, and calculation of *f*T _> MIC_ and PTA was performed using Pmetrics version 1.5.2 (Laboratory of Applied Pharmacokinetics and Bioinformatics, Los Angeles, CA, USA) [28], Intel Visual Fortran Compiler XE 14.0 (Santa Clara, CA, USA), RStudio (version 1.1.456), and R (version 3.5.1). The raw VPC data were imported from Pmetrics into PsN (version 4.6.0) using the Pirana interface [27] to generate VPCs with the same layout as NONMEM. VPC plots were subsequently created using XPose (version 4.6.1) within RStudio (version 1.1.456).

## 3. Results

### 3.1. Population

Demographic and clinical characteristics of the population (*n* = 26) used to build the popPK models and of the validation population (*n* = 19) are summarized in Table 1. None of the patients received continuous renal replacement therapy (CRRT). The medians of the APACHE II score and age were higher in the validation group compared to the modeling population. The median eGFR was lower in the validation group. Six validation subjects had an absolute CKD-EPI eGFR lower than the minimum of 51 mL/min in the modeling group, while one validation subject had an eGFR above the maximum of 172 mL/min in the modeling population. The other characteristics were comparable between the two groups.

### 3.2. External Validation

**Figure 1:**
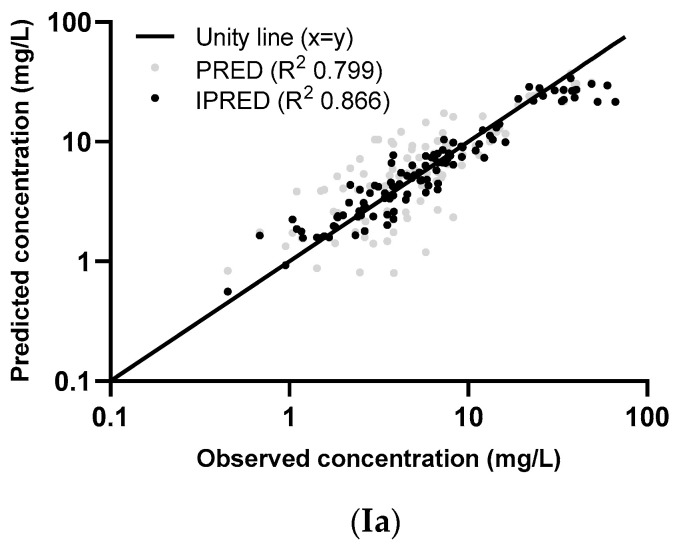

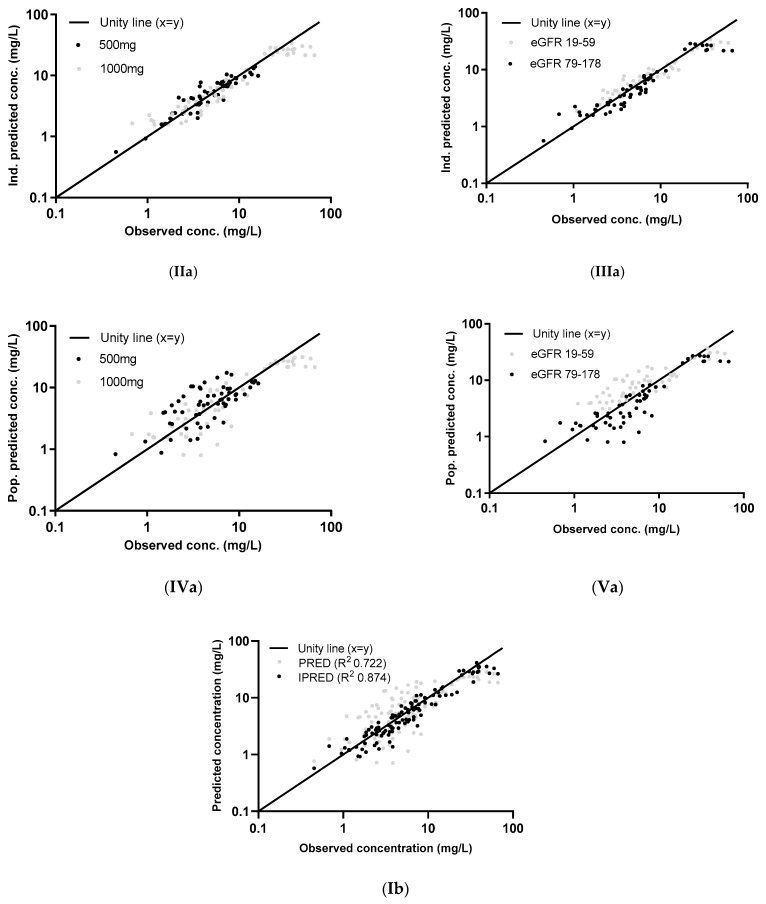

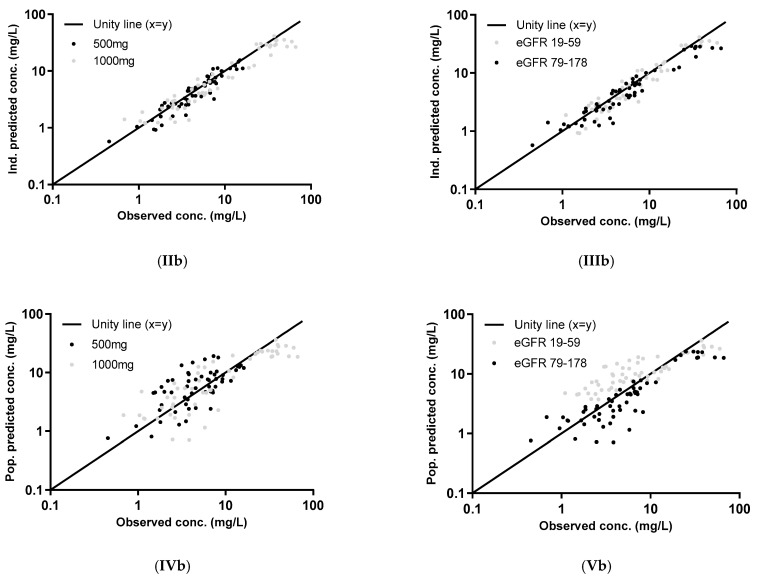
Individual (I [IPRED], II, and III) and population (I (PRED), IV, and V) concentrations, predicted using the parametric model (**a**) and the nonparametric model (**b**), plotted against the observed concentrations of the external dataset. The two dose groups, 500 mg and 1000 mg, are differentiated in graphs II and IV and two eGFR groups (measured by the CKD-EPI unadjusted for BSA) in graphs III and V. The log-transformed concentrations of the parametric model (**a**) are back transformed for an easier comparison with the untransformed concentrations in the figures of the nonparametric model (**b**).

**Figure 2:**
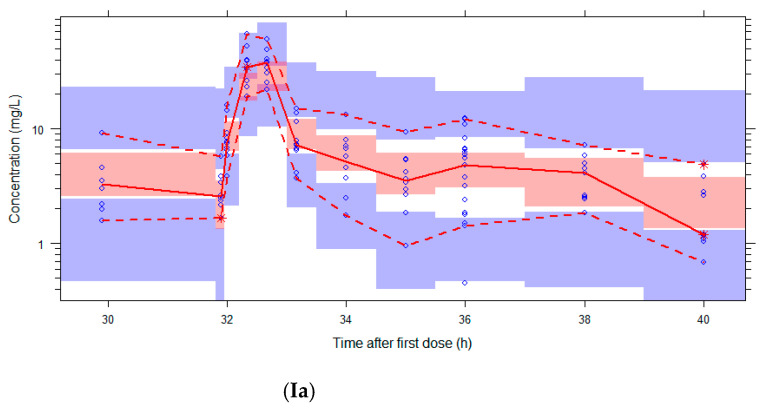

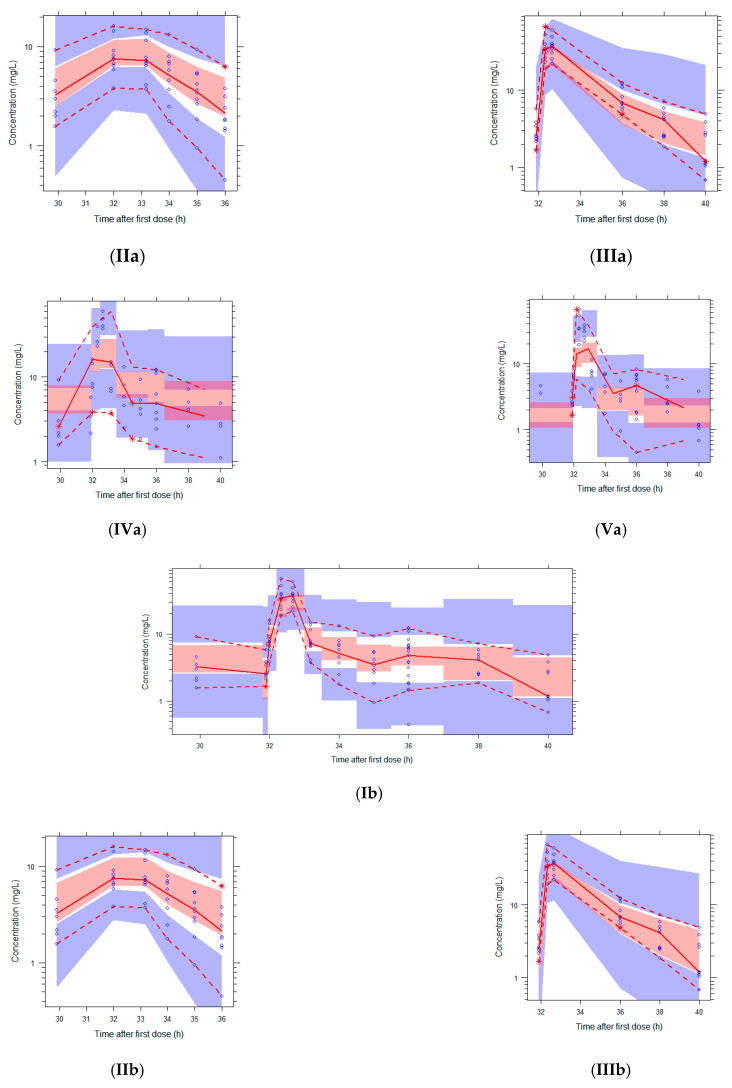

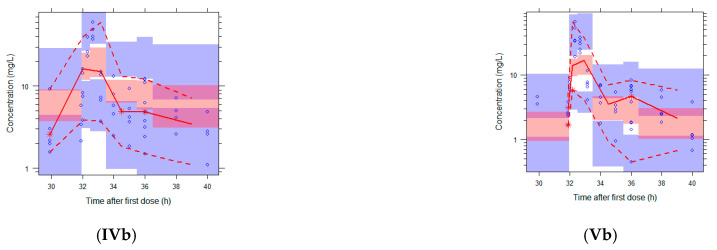
Visual Predictive Checks (VPCs) of both models using the external validation database. Circles: observed concentrations. Upper, middle, and lower lines: 95th, 50th, and 5th percentile of observations. Shaded areas: 95%CI of the corresponding percentiles of predictions. I: both dose regimens, II: 500 mg, III: 1000 mg, IV: eGFR 20–59 mL/min, V: eGFR 79–178 mL/min. The log-transformed concentrations of the parametric model (**a**) are back transformed for an easier comparison with the untransformed concentrations in the figures of the nonparametric model (**b**).

The graphs of individual and population predicted concentrations plotted against the observed concentrations of the external dataset (Figure 1) were comparable for the two models. Both models showed good predictive performance for 500 mg as well as 1000 mg, except for concentrations higher than approximately 20 mg/L for the 1000 mg dose (see also the visual predictive checks (VPCs) in Figure 2). The same deviation of the peak concentration was still shown in VPCs without samples during infusion (data not shown).

The VPCs with eGFR stratification on three groups were unclear due to the small group size (data not shown). Stratification on the two eGFR groups was better, but the VPC plots are still not optimal due to the different sampling times of both dosing groups, which were equally distributed among the eGFR groups (see Figure 2). The individual plots in Figure 1 and Figure 2, with stratification on the two eGFR groups, show that the predictive performance for eGFRs of 19–59 mL/min (*n* = 9) was worse than for eGFRs of 79–178 mL/min (*n* = 10). The VPCs show that both models predict too high concentrations for the trough levels of the low eGFR group. The median relative prediction error (Table 2) was higher for trough levels in the low eGFR group (parametric: 83% and nonparametric: 88%) than in the high eGFR group (−24% and −19%). These prediction errors were comparable for the 500 mg and 1000 mg in each eGFR group (data not shown).

The median prediction error and median relative prediction error after 1000 simulations were similar to the single external validation for both models (Table 2), although the 97.5–2.5% range is larger after the simulations using the nonparametric model. The wider distribution of the nonparametric model is also shown in the VPCs in Figure 2.

The proportion of observations between the 5th and 95th simulated percentiles in the VPCs in Figure 2 are: 96% (Ia), 95% (IIa), 94% (IIIa), 81% (IVa), 88% (Va), 96% (Ib), 97% (IIb), 96% (IIIb), 81% (IVb), and 95% (Vb).

### 3.3. Simulations

The highest MICs with a probability of target attainment (PTA) >97.5% for a target of 50% and 100% *f*T _> MIC_ attained by several imipenem dosing regimens and eGFR values of 150, 120, 90 mL/min are shown in Table 3 for both models. Fifty percent of the MICs calculated using the parametric model were equal to those calculated by the nonparametric model and the other half of the MICs were lower for the nonparametric model. The PTAs for the full MIC profile from 0.015 to 64 mg/L are shown in Supplementary Table S2.

## 4. Discussion

The external validation of parametric and nonparametric popPK models of imipenem in critically ill patients showed that the predictive performance of both models was sufficient in patients with high eGFRs (79–178 mL/min). However, the models could not be extrapolated to patients with lower eGFRs, as they were hardly included in the population used to build the model. The PTA simulations using both models were therefore performed for eGFR ≥ 90 mL/min only. Fifty percent of the PTA calculations resulted in similar MICs for both models, while the other half of the simulations resulted in lower MICs for the nonparametric model.

Our external validation simulations showed that the median PE and RPE were comparable for both models, although the ranges of the PE and RPE were wider for the nonparametric model. This was also shown in the VPCs and can be explained by the higher BSV in the nonparametric model. In contrast to the external validation simulations, the original external validation (without simulations) showed higher medians of the PE and RPE for the nonparametric model. However, the mean PEs (−1.9 mg/L parametric and –1.8 mg/L nonparametric) were similar. Given the similar external validation simulation results, this deviation of median PEs and RPEs might be caused by the small study size.

The poor predictive performance for low eGFRs can be explained by the paucity of subjects with renal impairment in the modeling population. Only 1 of 26 patients in the modeling population (*n* = 26) had an eGFR lower than 90 mL/min, while this applied to 12 of 19 patients in the validation population. After the external validation showed that both popPK models could not be extrapolated to low eGFRs, we decided to perform the PTA simulations for eGFR ≥ 90 mL/min only, deviating from the original plan to also simulate for lower eGFRs. The cut-off of 90 mL/min was chosen from a practical point of view, in line with the package inserts [22,26] instead of the eGFR ranges from the VPCs (19–59 and 79–178 mL/min), which were chosen to create two equal groups. The popPK models are still applicable to a high proportion of critically ill patients. Augmented renal clearance (defined as increased renal elimination of circulating solutes and drugs as compared with normal baseline [29]) has been reported in approximately 30–65% of critically ill patients [30].

The VPCs stratified on dose showed for both models a good predictive performance for the 500 and 1000 mg regimens, except for peak concentrations (C_max_) for the 1000 mg dose. This dose could not be tested during popPK modeling because the modeling population only used 500 mg. The higher than predicted C_max_ is not likely to be explained by non-linear PK [22,31]. The most probable reasons for the C_max_ deviation are the critical timing of the peak samples and the variable PK in critically ill patients [32], which is also shown by others [31,33,34,35]. However, it is important to realize that, instead of C_max_, the trough level is relevant for the targets of 50–100% *f*T _> MIC_. Importantly, the simulations did not show different prediction errors for trough levels after 500 mg and 1000 mg. Therefore, we decided to include 1000 mg dose regimens in the PTA simulations.

Our external validation findings emphasise the importance of such a validation when popPK models are used to optimize dosing strategies based on PTA simulations, or to individualize dosing by therapeutic drug monitoring software. PopPK model publications often not include external validation. A survey of the literature revealed that only for 7% of popPK models published between 2002 and 2004 (*n* = 324) was an external evaluation performed [36]. To our knowledge, a more recent survey does not exist.

Our simulations showed that 50% of the PTA results were comparable for both models, while the other half resulted in lower MICs for the nonparametric model, although the majority (7/9 = 78%) differed by only one dilution. The lower MICs could be caused by a higher estimated BSV of the popPK parameter values in the nonparametric model, leading to a wider range of concentrations. It is impossible to judge which of the models represents the “truth”. The parametric model could have included too little variability or the nonparametric model could be too flexible. Probably, the truth is somewhere in between. We compared our simulation results with other published M&S studies of imipenem in critically ill patients, of which one was based on a parametric model [15] and two on nonparametric models [16,17]. Despite differences in parameter estimates, our finding of higher MICs with the parametric model was confirmed by these papers [15,16,17]. Similar to two studies [15,17], we showed that it is difficult to reach high MICs from 1 mg/L with an increased target of 100% *f*T _> MIC_, which confirms again that more prospective studies about the required target of beta-lactams in critically ill patients are needed. The regression analysis of the original study [20], with four beta-lactams from which we analysed a subgroup, did not find a significant association between clinical failure and trough levels below 2 mg/L, indicating that an elevated target of 100% *f*T _> MIC_ might not be necessary in this population. Even a larger study was underpowered to find a significant association between clinical failure and troughs below 2 mg/L [19]. Importantly, the latter study [19] proved that fear about toxicity at high doses of 3–4 g/day is unnecessary, as patients receiving these doses did not have increased toxicity compared to the standard dose of 2 g/day.

Based on the 50% *f*T _> MIC_ target simulations, we conclude that a high dose of 1000 mg q6h is required to maximize the probability to reach MICs of 2 mg/L (e.g., for *Enterobacterales* [37]) in critically ill patients with eGFRs of 90–120 mL/min, although the PTAs using the nonparametric model were below the 97.5% cut-off but still above 90% (see Supplementary Table S2). This is in line with the American prescribing information [26]. However, they recommend this dosing regimen also for MICs of 4 mg/L (e.g., for *Pseudomonas aeruginosa*), similar to the European product characteristics [22], although these brochures are of course not specifically dedicated to critically ill patients. Considering our simulation results, it is difficult to give dosing recommendations for MICs of 4 mg/L, and also for higher MICs of 2 mg/L and eGFRs of 150 mL/min, because the PTA values differ largely per modeling approach. For example, for 1000 mg q6h, eGFR 90, 50% *f*T _> MIC_, and MIC 4 mg/L, the PTA was 90% for the parametric model and 63% for the nonparametric model (see also Supplementary Table S2). Dosing regimens may look acceptable following the parametric model, while the nonparametric model might plead for increased dosing. As previously stated, the truth may be somewhere in between. One of the objectives of our study was to determine which of the two previously described imipenem models delivers the best Bayesian posterior estimates to predict the imipenem concentrations in an external independent database. It was not possible to assign a winner, because the external predictive performance of both models was adequate. However, as the dosing simulations based on the models show different results, more research on this topic is clearly needed. Until now, we recommend all readers of M&S papers to be aware of the consequences of the chosen modeling approach before implementing the dosing recommendations of these papers in clinical practice.

Few studies comparing parametric and nonparametric M&S are available in the literature. Precluding the studies with currently outdated software [38,39,40,41], we found eight comparison studies [42,43,44,45,46,47,48,49]. For three of these studies, the parameters of both models could not be compared due to a different model structure [42,43] or unreported values [46]. The majority of the remaining five comparison studies showed comparable parameter estimates of both models [45,47,48,49], although the BSV of the parametric estimates was often higher for the nonparametric model [44,45,47], similar to our findings. The three comparison studies that performed an external validation of both models concluded that the nonparametric models provided the lowest relative prediction error (RPE) for concentrations [46] and area under the curve [43,44], although for one of the latter studies [44], the RPE of the concentrations was similar. Our external validation showed a comparable RPE for both models after simulations, reflecting a good predictive performance of both models. PTA calculations using both modeling approaches were only performed by one previous study [48], which concluded that the PTA versus MIC profiles (based on 10,000 simulations) were similar. This seems to be caused by the similar parameter estimates and BSV of these models. Contrary to the latter study, our PTA simulations show different results for both modeling approaches, which could be explained by the higher BSV of our nonparametric model.

This paper has a few limitations. The main limitation is that the modeling population did not include 1000 mg dose regimens as well as patients with impaired renal function. A drawback of the validation population was that only 12 patients matched the eGFR range of the modeling population (51–172 mL/min). Another limitation is that the simulations were performed with a fixed value of protein binding because only total drug concentrations were available. However, the consequences seem to be low given the small protein binding of 20% [22]. 

## 5. Conclusions

The external predictive performance of parametric and nonparametric popPK models of imipenem in critically ill patients was adequate for subjects with high eGFRs, but insufficient for low eGFRs. This was explained by a paucity of subjects with renal impairment in the modeling population. External validation of popPK models is important to test the possibility of extrapolation to other populations. The PTA simulations of both models indicated that 1000 mg q6h is suitable to reach MICs of 2 mg/L in critically ill patients with eGFRs of 90–120 mL/min. However, for MICs of 2 mg/L and an eGFR of 150 mL/min, and for MICs of 4 mg/L, dosing recommendations could not be given because the PTA values differed largely per modeling approach. The consequences of the different modeling approaches in clinical practice should be further investigated.

## Figures and Tables

**Table 1:** Demographic and clinical characteristics of the population (*n* = 26) used to build the popPK models and of the validation population (*n* = 19). APACHE, Acute Physiology and Chronic Health Evaluation; eGFR, estimated Glomerular Filtration Range; CKD-EPI, Chronic Kidney Disease Epidemiology Collaboration; BMI, Body Mass Index; BSA, Body Surface Area.

Parameter	Modeling Population	Validation Population
Male, *n* (%)	18 (69)	14 (74)
APACHE II score, median (range)	22 (7–35)	26 (13–42)
Age (years), median (range)	51 (25–59)	64 (26–90)
Creatinine at inclusion (μmol/L), median (range)	59 (28–108)	98 (44–235)
eGFR CKD-EPI at inclusion (ml/min/1.73 m^2^), median (range)	116 (50–143)	73 (20–145)
eGFR absolute CKD-EPI at inclusion, unadjusted for BSA (ml/min), median (range)	119 (51–172)	79 (19–178)
Height (cm), median (range)	175 (155–190)	170 (150–190)
Total bodyweight (kg), median (range)	75 (50–107)	78 (45–110)
BMI (kg/m^2^), median (range)	25 (18–35)	28 (18–34)
BSA (m^2^), median (range)	1.89 (1.51–2.23)	1.92 (1.40–2.29)
Presumed infection, *n* (%)		
Respiratory tract infection	16 (62)	19 (100)
Intra-abdominal infection	4 (15)	-
Bloodstream infection	3 (12)	-
Surgical site infection	1 (4)	-
Meningitis	1 (4)	-
Gynecological infection	1 (4)	-

**Table 2:** Prediction errors of the parametric and nonparametric popPK models using the external validation database (111 concentrations). The prediction errors were also calculated after 1000 simulations (111.000 concentrations) of both models. In the last 4 columns, a selection of the simulations (trough levels only) per eGFR group are shown. PE = prediction error (mg/L) = individual predicted concentration—observed concentration. RPE = relative prediction error (%) = prediction error/observed concentration.

KERRYPNX	External Database		Simulations		Simulations (Selection)		Simulations (Selection)	
	111 Concentrations		1000 × 111 Concentrations		1000 × 17 trough eGFR19-59		1000 × 18 trough eGFR79-178	
**Parametric**	**PE (mg/L)**	**RPE (%)**	**PE (mg/L)**	**RPE (%)**	**PE (mg/L)**	**RPE (%)**	**PE (mg/L)**	**RPE (%)**
97.5%	3.83	105	8.97	252	9.74	360	2.03	225
75%	0.61	19	1.97	56	3.92	167	0.38	31
50%	−0.02	−1	−0.04	−1	2.13	83	−0.50	−24
25%	−1.52	−20	−2.20	−31	0.72	23	−1.64	−53
2.5%	−30.55	−52	−28.63	−74	−3.16	−41	−3.15	−82
**Nonparametric**	**PE (mg/L)**	**RPE (%)**	**PE (mg/L)**	**RPE (%)**	**PE (mg/L)**	**RPE (%)**	**PE (mg/L)**	**RPE (%)**
97.5%	3.89	54	30.68	594	28.96	996	7.08	564
75%	0.51	15	3.22	83	5.66	221	0.80	58
50%	−0.43	−9	0.02	0.5	2.24	88	−0.33	−19
25%	−1.74	−29	−2.47	−39	0.32	11	−1.56	−56
2.5%	−25.99	−58	−24.77	−79	−4.15	−63	−3.36	−91

**Table 3:** The highest MIC for which a probability of target attainment (PTA) of 97.5% is reached at targets of 50% and 100% *f*T > MIC by several imipenem dosing regimens and eGFR values (measured by the CKD-EPI equation unadjusted for BSA) of 150, 120, and 90 mL/min. The PTAs were calculated by Monte Carlo simulations (*n* = 5000) using parametric and nonparametric popPK models.

eGFR (ml/min)	Dose Regimen	Target *f*T _> MIC_	Highest MIC (mg/L) with PTA > 97.5%	
			Parametric	Nonparametric
150	500 mg q6h	100%	0.125	0.06
	1000 mg q8h	100%	0.125	0.03
	1000 mg q6h	100%	0.25	0.125
	500 mg q6h	50%	0.5	0.25
	1000 mg q8h	50%	0.5	0.5
	1000 mg q6h	50%	1	1
120	500 mg q6h	100%	0.125	0.125
	1000 mg q8h	100%	0.25	0.06
	1000 mg q6h	100%	0.25	0.25
	500 mg q6h	50%	0.5	0.5
	1000 mg q8h	50%	1	0.5
	1000 mg q6h	50%	2	1
90	500 mg q6h	100%	0.25	0.25
	1000 mg q8h	100%	0.25	0.25
	1000 mg q6h	100%	0.5	0.5
	500 mg q6h	50%	1	0.5
	1000 mg q8h	50%	1	1
	1000 mg q6h	50%	2	1

## Data Availability

The data presented in this study are available on request from the corresponding author. The data are not publicly available due to controlled access requirements for clinical trial data.

## Supplementary Materials

The following are available online at https://www.mdpi.com/article/10.3390/pharmaceutics13122170/s1, Table S1: Population parameter estimates; Table S2: Probabilities of target attainment (PTA) simulations.
