# Supplementary material for: Parametric and Nonparametric Population Pharmacokinetic Models to Assess Probability of Target Attainment of Imipenem Concentrations in Critically Ill Patients
Source: Pharmaceutics. 2021 Dec 16;13(12):2170. doi: 10.3390/pharmaceutics13122170 (PMC8709176; doi:10.3390/pharmaceutics13122170)
Supplement: Supplementary file 1 [file pharmaceutics-13-02170-s001.zip › pharmaceutics-1478535-supplementary.pdf]

pharmaceutics

MDPI

Supplementary Materials: Parametric and Nonparametric Population Pharmacokinetic Models to Assess Probability of Target Attainment of Imipenem Concentrations in Critically Ill Patients

Femke de Velde , Brenda C. M. de Winter, Michael N. Neely, Jan Strojil, Walter M. Yamada, Stephan Harbarth, Angela Huttner, Teun van Gelder, Birgit C. P. Koch, Anouk E. Muller
on behalf of the COMBACTE-NET Consortium †

Table S1. Population parameter estimates. V_c: central distribution volume, K_{cp}: rate constant from central to peripheral compartment, K_{pc}: rate constant from peripheral to central compartment, K_e: elimination rate constant, $K_e$cov: covariate effect on K_e, CV: coefficient of variation.

Parameter	NONMEM		Pmetrics	
	Parameter estimate	CV (%)	Mean parameter estimate	CV (%)
V_c (L)	29.6	-	31.1	42.6
K_{cp} (h^{-1})	0.166	-	0.374	81.2
K_{pc} (h^{-1})	0.195	-	0.495	72.0
K_e (h^{-1})	0.637	19.0	0.681	34.0
K_e(cov)	0.655	-	0.658	55.2
Parametric model (NONMEM): exponential error (mg/L)	0.348	-	N/A	-
Nonparametric model (Pmetrics): gamma (error model)	N/A	-	3.40	-

Table S2. Probabilities of target attainment (PTA) for targets of 50% and 100% $fT_{>MIC}$ attained by several imipenem dosing regimens and eGFR values (measured by the CKD-EPI equation unadjusted for BSA) of 150, 120 and 90 mL/min. The PTAs were calculated by Monte Carlo simulations (n = 5000) using parametric (**a**) and nonparametric (**b**) popPK models. The grey shaded cells indicate the highest MIC for which the PTA is higher than 97.5%.

Table S2a. PTA simulations using the parametric model.

eGFR (mL/min)	Dose regimen	Target $fT_{>MIC}$	PTA (%) at specified MIC (mg/L)												
			0.015	0.03	0.06	0.125	0.25	0.5	1	2	4	8	16	32	64
**150**	**500mg q6h**	100%	100	100	100	99	81	22	0	0	0	0	0	0	0
	1000mg q8h	100%	100	100	100	100	95	47	4	0	0	0	0	0	0
	1000mg q6h	100%	100	100	100	100	99	84	27	1	0	0	0	0	0
	500mg q6h	50%	100	100	100	100	100	100	84	15	0	0	0	0	0
	1000mg q8h	50%	100	100	100	100	100	100	91	40	1	0	0	0	0
	1000mg q6h	50%	100	100	100	100	100	100	100	94	32	0	0	0	0
120	500mg q6h	100%	100	100	100	100	94	50	3	0	0	0	0	0	0
	1000mg q8h	100%	100	100	100	100	99	76	15	0	0	0	0	0	0
	1000mg q6h	100%	100	100	100	100	100	96	58	5	0	0	0	0	0
	500mg q6h	50%	100	100	100	100	100	100	96	39	0	0	0	0	0
	1000mg q8h	50%	100	100	100	100	100	100	98	71	6	0	0	0	0
	1000mg q6h	50%	100	100	100	100	100	100	100	99	63	0	0	0	0
90	500mg q6h	100%	100	100	100	100	100	84	21	0	0	0	0	0	0
	1000mg q8h	100%	100	100	100	100	100	96	50	2	0	0	0	0	0
	1000mg q6h	100%	100	100	100	100	100	100	88	26	0	0	0	0	0
	500mg q6h	50%	100	100	100	100	100	100	100	77	1	0	0	0	0
	1000mg q8h	50%	100	100	100	100	100	100	100	93	28	0	0	0	0

	1000mg q6h	50%	100	100	100	100	100	100	100	100	90	4	0	0	0

Table S2b. PTA simulations using the nonparametric model.

eGFR (mL/min)	Dose regimen	Target $fT_{>MIC}$	PTA (%) at specified MIC (mg/L)												
			**0.015**	**0.03**	**0.06**	**0.125**	**0.25**	**0.5**	**1**	**2**	**4**	**8**	**16**	**32**	**64**
150	500mg q6h	100%	100	99	98	92	78	47	13	2	1	0	0	0	0
	1000mg q8h	100%	99	99	96	90	79	56	24	4	1	0	0	0	0
	1000mg q6h	100%	100	100	100	98	94	81	39	6	1	0	0	0	0
	500mg q6h	50%	100	100	100	100	100	96	74	25	5	1	0	0	0
	1000mg q8h	50%	100	100	100	100	100	98	87	49	13	2	0	0	0
	1000mg q6h	50%	100	100	100	100	100	100	98	81	31	5	1	0	0
120	500mg q6h	100%	100	100	100	98	91	64	20	3	1	0	0	0	0
	1000mg q8h	100%	100	100	99	97	90	72	35	6	1	0	0	0	0
	1000mg q6h	100%	100	100	100	100	99	93	52	10	2	1	0	0	0
	500mg q6h	50%	100	100	100	100	100	99	88	39	7	2	1	0	0
	1000mg q8h	50%	100	100	100	100	100	100	96	67	20	3	1	0	0
	1000mg q6h	50%	100	100	100	100	100	100	99	91	44	8	1	1	0
90	500mg q6h	100%	100	100	100	100	98	87	36	6	2	1	0	0	0
	1000mg q8h	100%	100	100	100	100	99	91	57	11	2	1	0	0	0
	1000mg q6h	100%	100	100	100	100	100	99	67	15	4	1	0	0	0
	500mg q6h	50%	100	100	100	100	100	100	95	59	13	3	1	0	0
	1000mg q8h	50%	100	100	100	100	100	100	98	85	36	6	1	0	0
	1000mg q6h	50%	100	100	100	100	100	100	100	97	63	15	3	1	0
